# Correction: Cohort profile: Genetic data in the German Socio-Economic Panel Innovation Sample (SOEP-G)

**DOI:** 10.1371/journal.pone.0303620

**Published:** 2024-05-09

**Authors:** The PLOS ONE Staff

## Notice of Republication

This article was republished on April 17, 2024, to correct an error in the author list: David Richter was erroneously displayed as David Richte. The publisher apologizes for this error. Please download this article again to view the correct version. The originally published, uncorrected article and the republished, corrected article are provided here for reference.

## Supporting information

S1 File Originally published, uncorrected article. (PDF)

S2 File Republished, corrected article. (PDF)

## References

[1] Koellinger PD, Okbay A, Kweon H, Schweinert A, Linnér RK, Goebel J, et al. (2023) Cohort profile: Genetic data in the German Socio-Economic Panel Innovation Sample (SOEP-G). PLoS ONE 18(11): e0294896. doi: 10.1371/journal.pone.0294896 38019829 PMC10686514

