# Supplementary figures and images for: Cohort profile: Genetic data in the German Socio-Economic Panel Innovation Sample (SOEP-G)
Source: PLoS One. 2023 Nov 29;18(11):e0294896. doi: 10.1371/journal.pone.0294896 (PMC10686514; doi:10.1371/journal.pone.0294896)

**S1 Fig.** Histogram of minor allele frequencies of genotyped autosomal biallelic SNPs

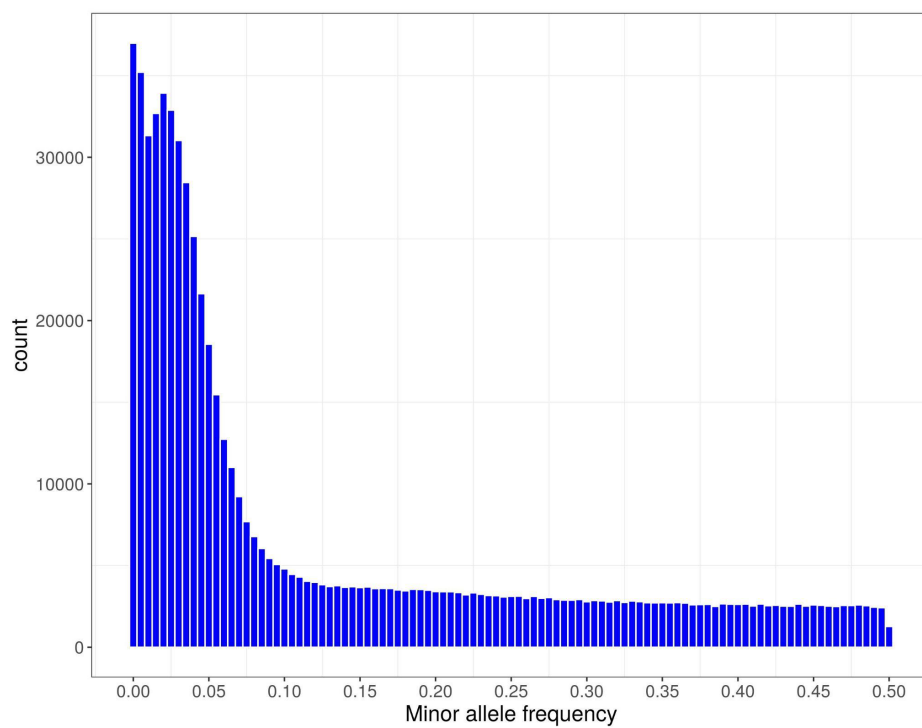

Supplement: S1 Fig — (PDF) [file pone.0294896.s001.pdf]

**S3 Fig.** Binned scatter plot of SNP missing rates over minor allele frequencies

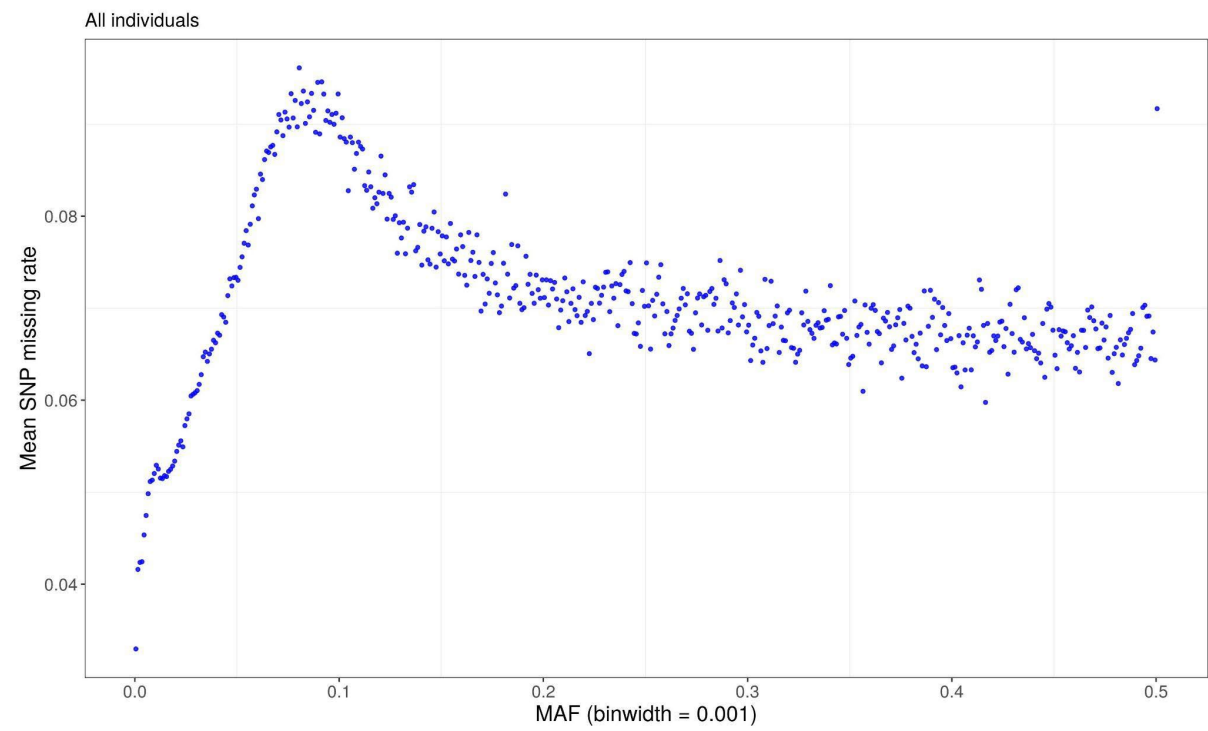

Supplement: S3 Fig — (PDF) [file pone.0294896.s003.pdf]

**S4 Fig.** Histogram of mean sample call rates by interviewer

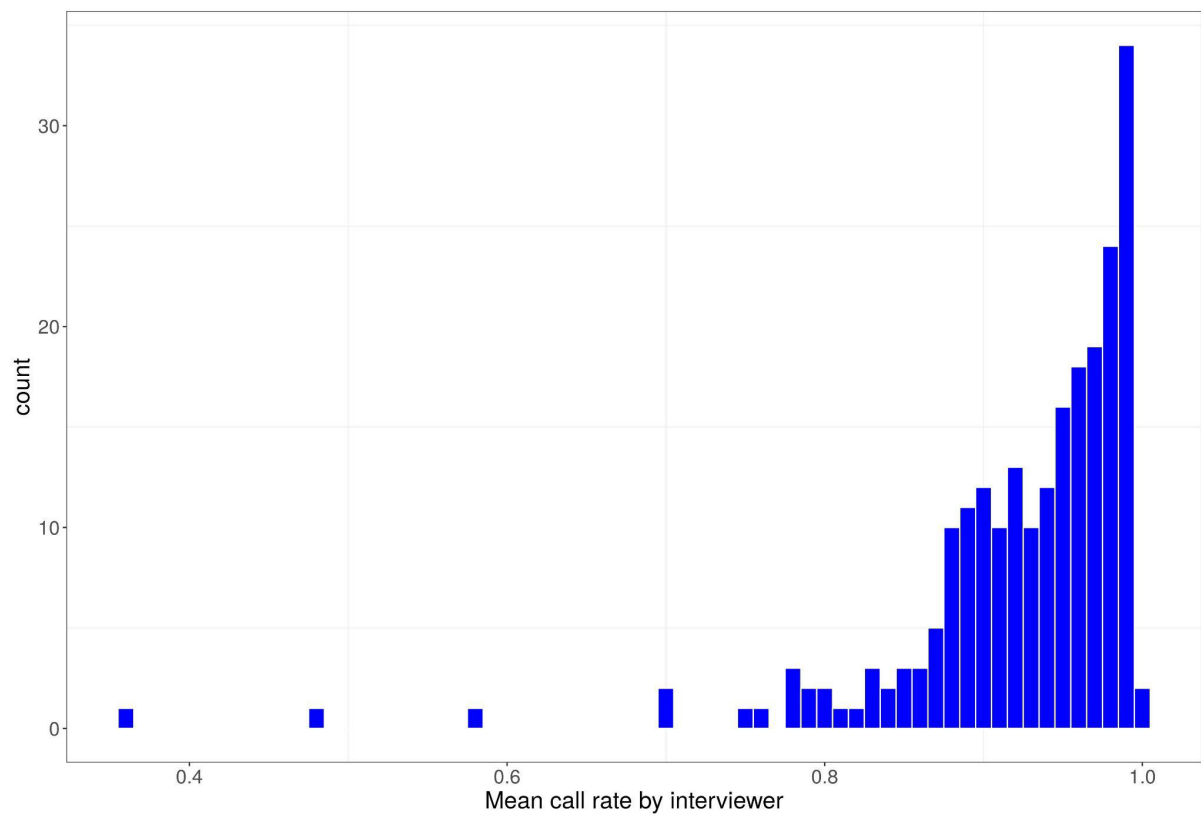

Supplement: S4 Fig — (PDF) [file pone.0294896.s004.pdf]

**S5 Fig.** Homozygosity / Heterozygosity outliers

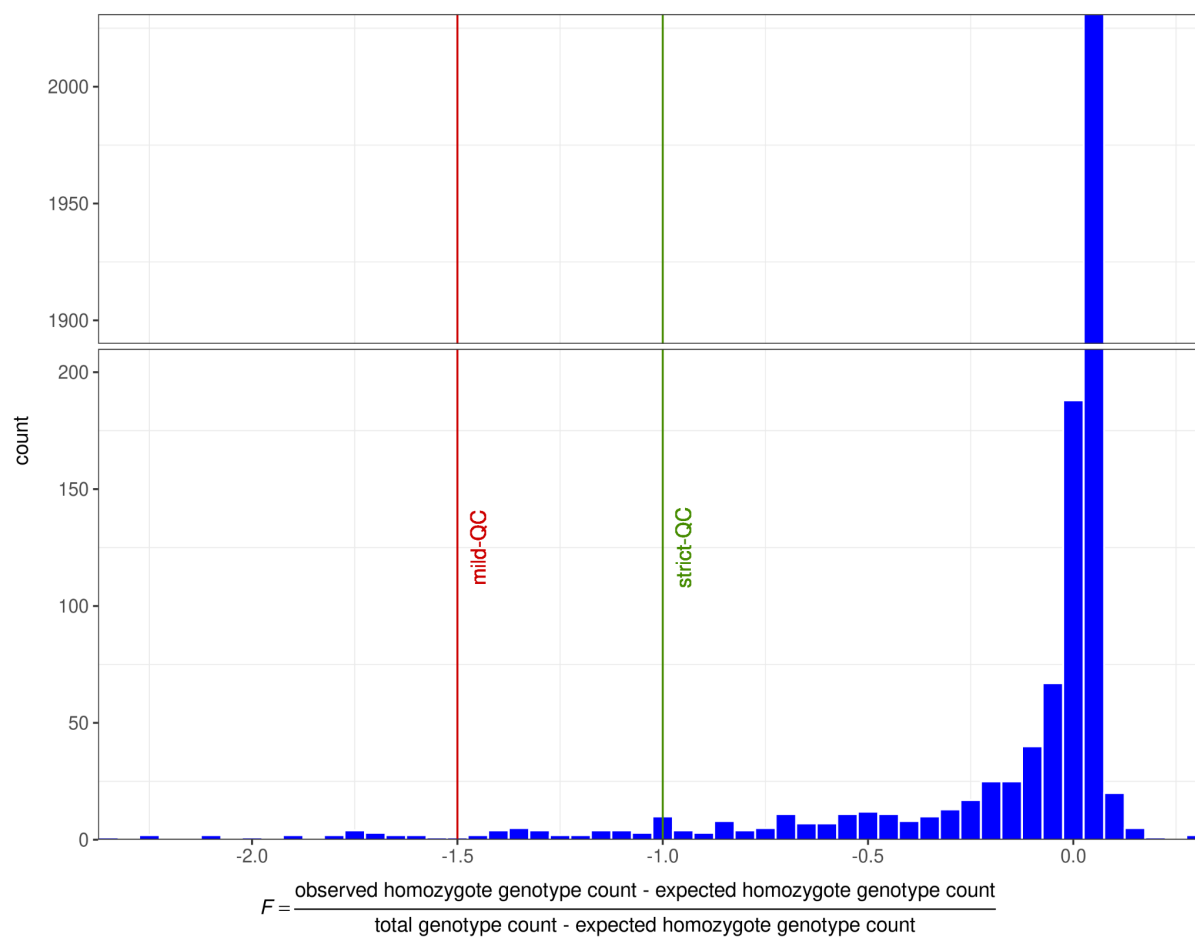

Supplement: S5 Fig — (PDF) [file pone.0294896.s005.pdf]

**S6 Fig.** Pre-imputation (mild-)QC - Ancestry filtering

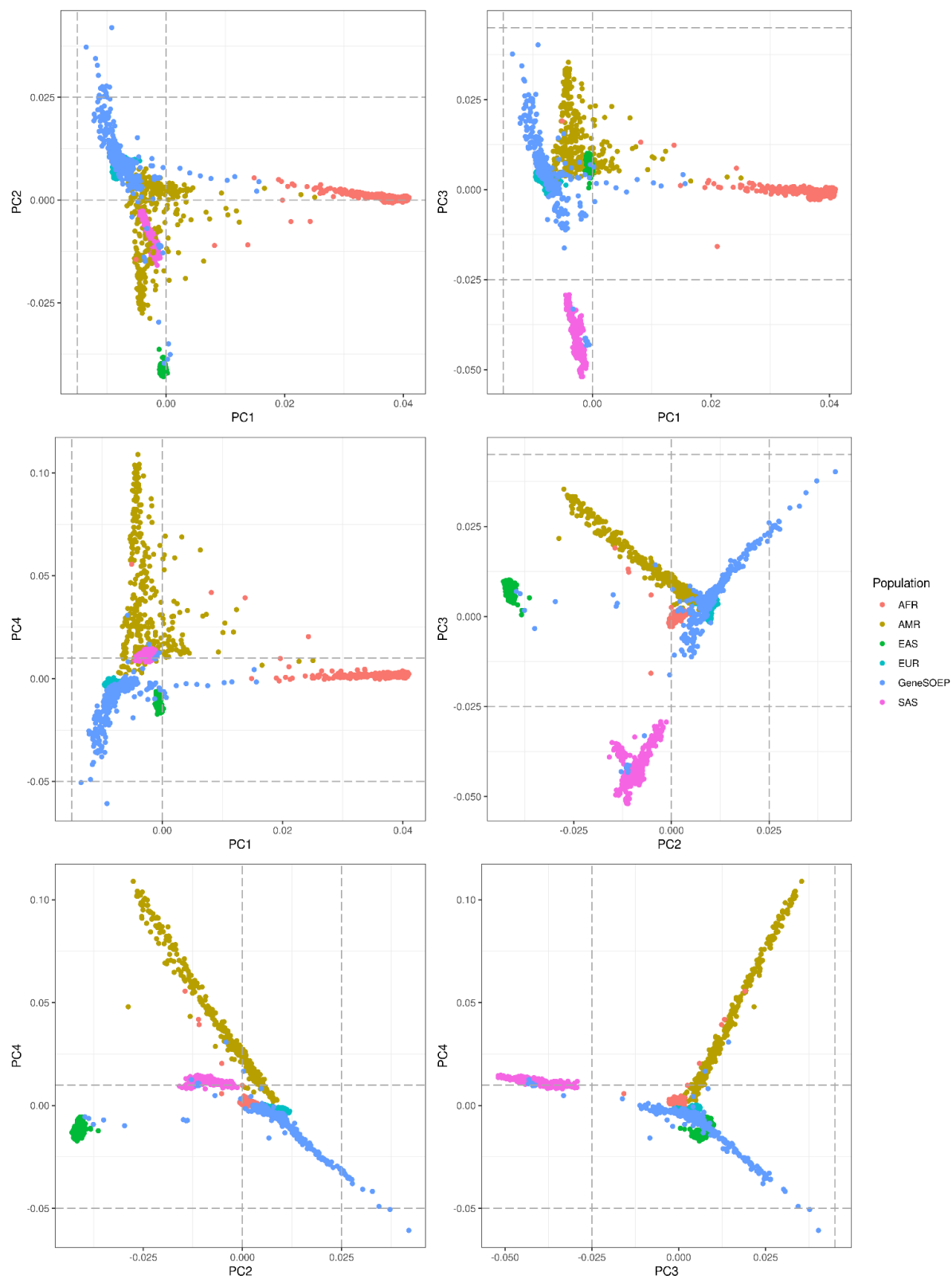

Supplement: S6 Fig — (PDF) [file pone.0294896.s006.pdf]

**S7 Fig.** Imputed data MAF distribution

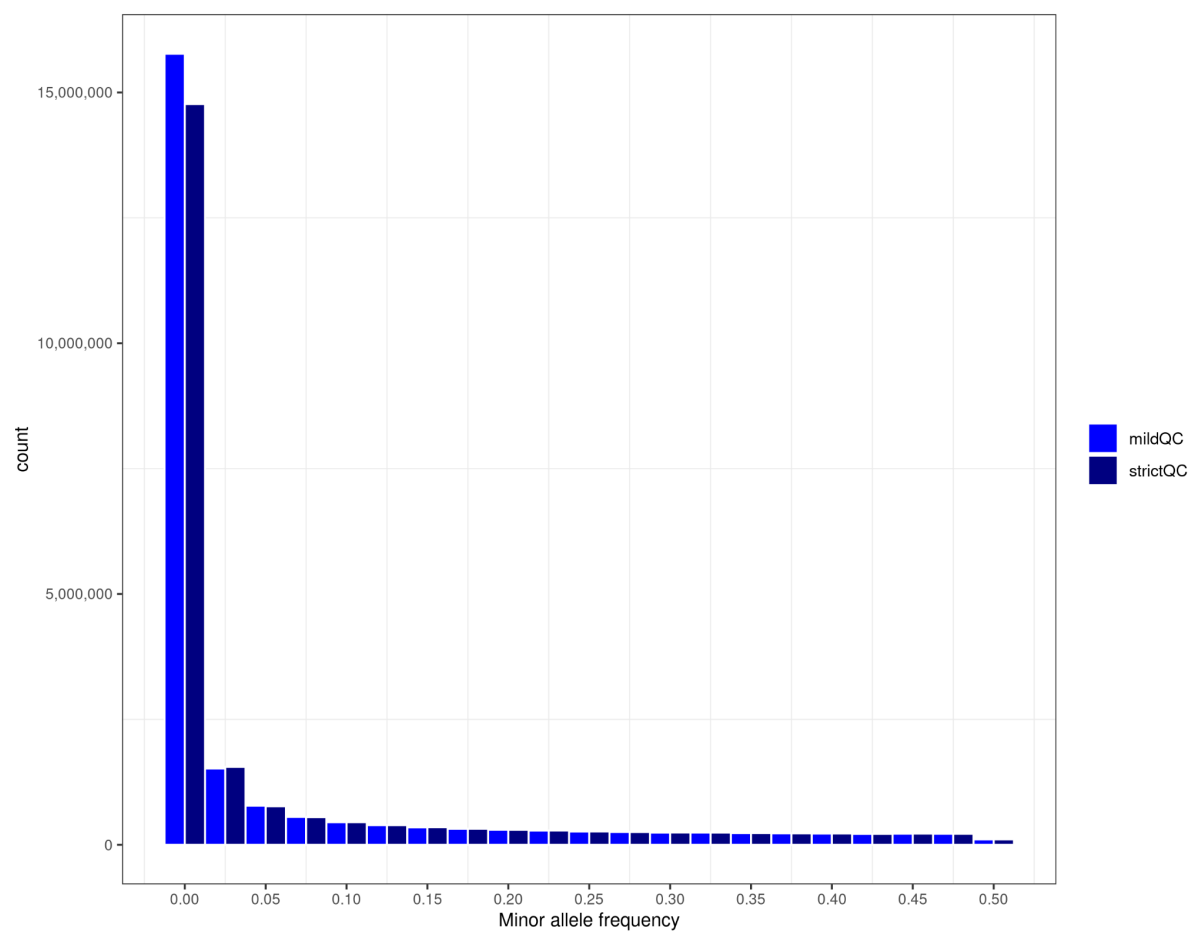

Supplement: S7 Fig — (PDF) [file pone.0294896.s007.pdf]

**S8 Fig.** Imputation accuracy distribution

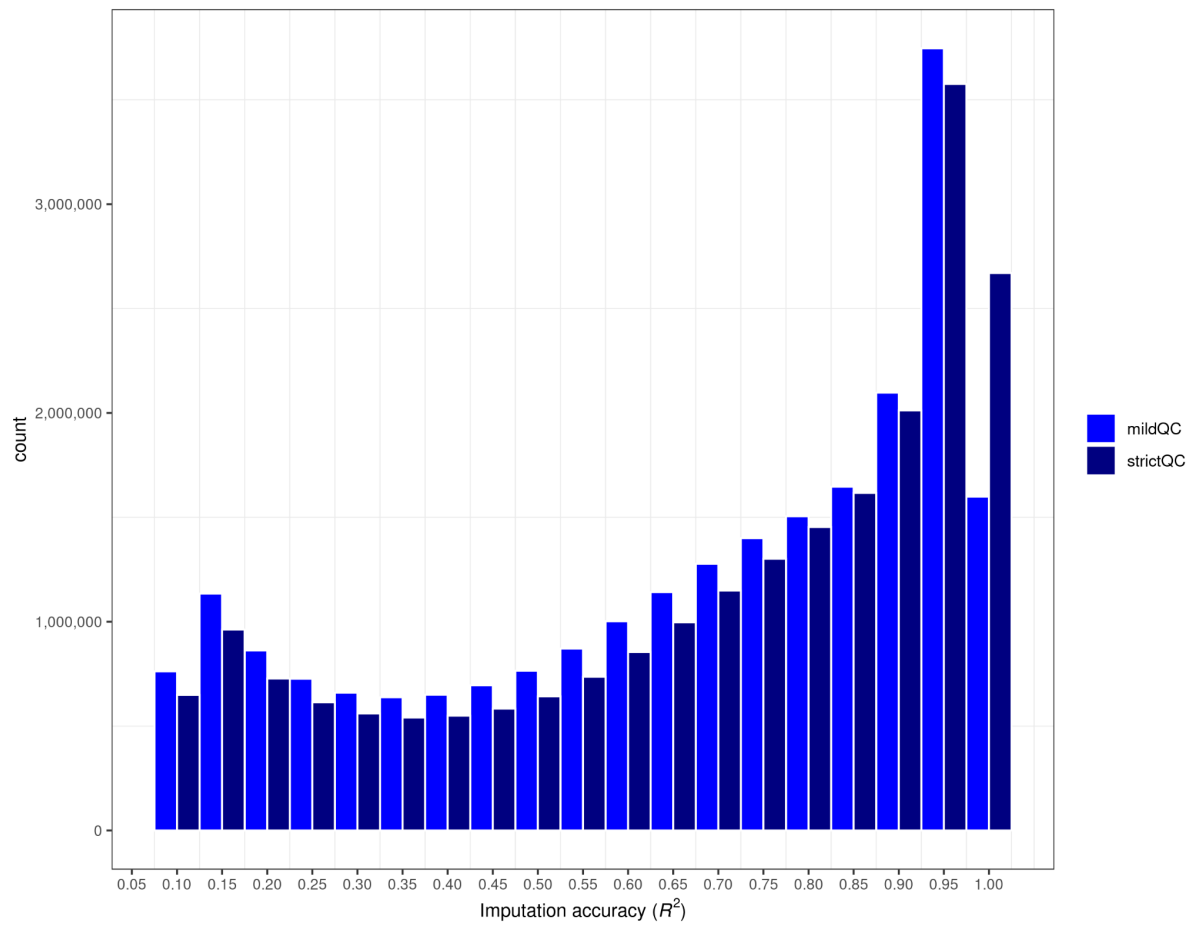

Supplement: S8 Fig — (PDF) [file pone.0294896.s008.pdf]

**S9 Fig.** Mean imputation accuracy by MAF

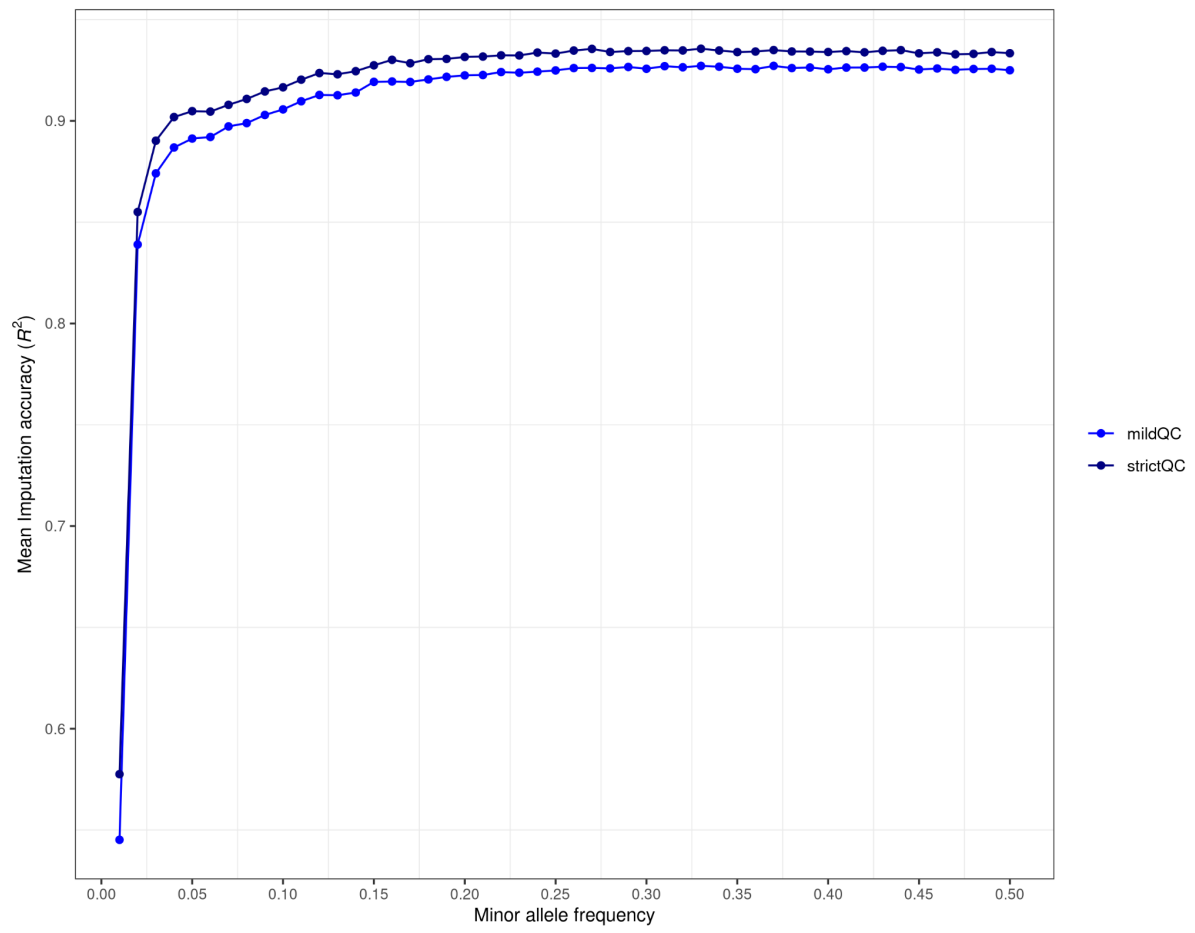

Supplement: S9 Fig — (PDF) [file pone.0294896.s009.pdf]

**S10 Fig.** Post-imputation mild-QC - Ancestry filtering

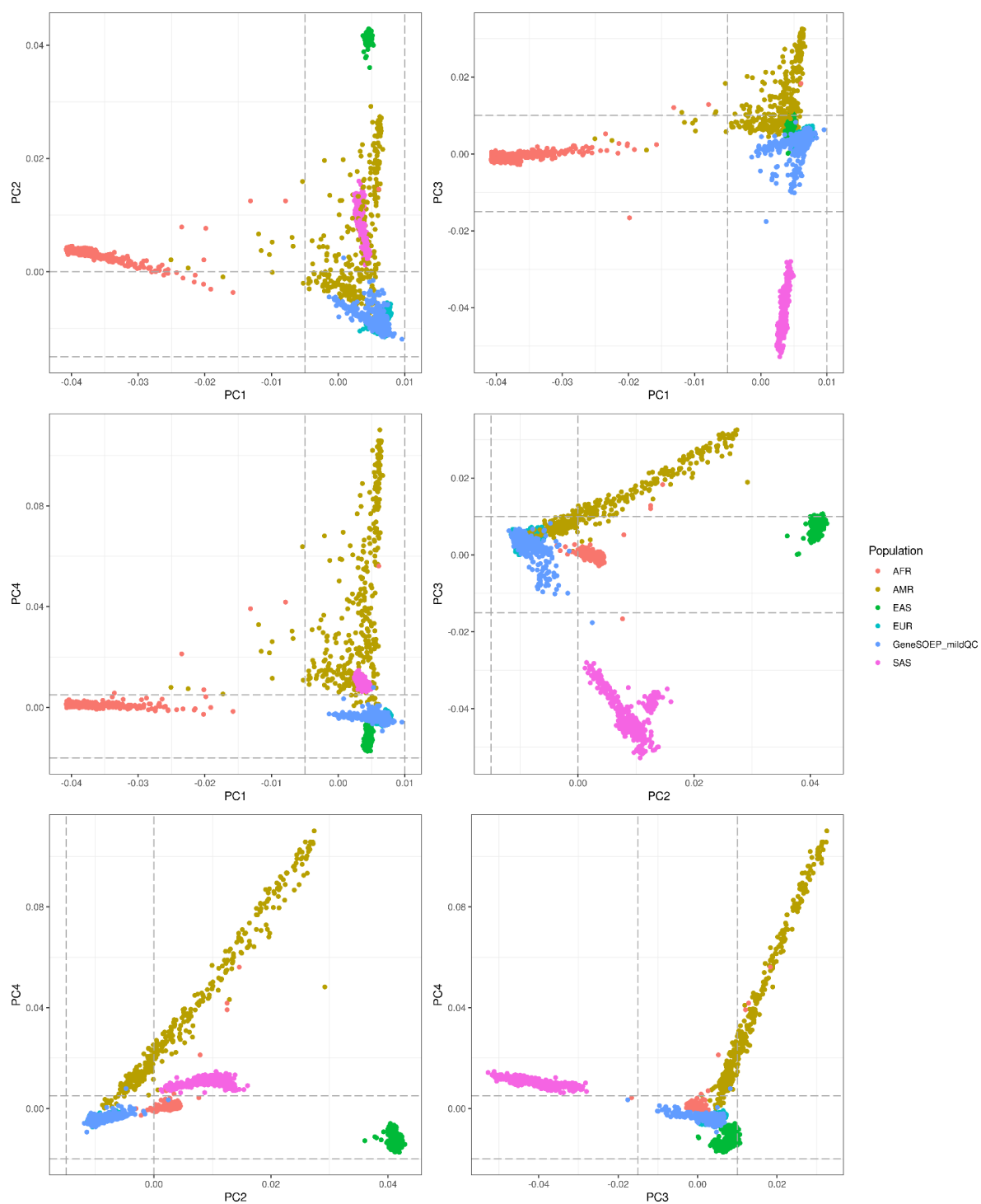

Supplement: S10 Fig — (PDF) [file pone.0294896.s010.pdf]

**S11 Fig.** Post-imputation strict-QC - Ancestry filtering

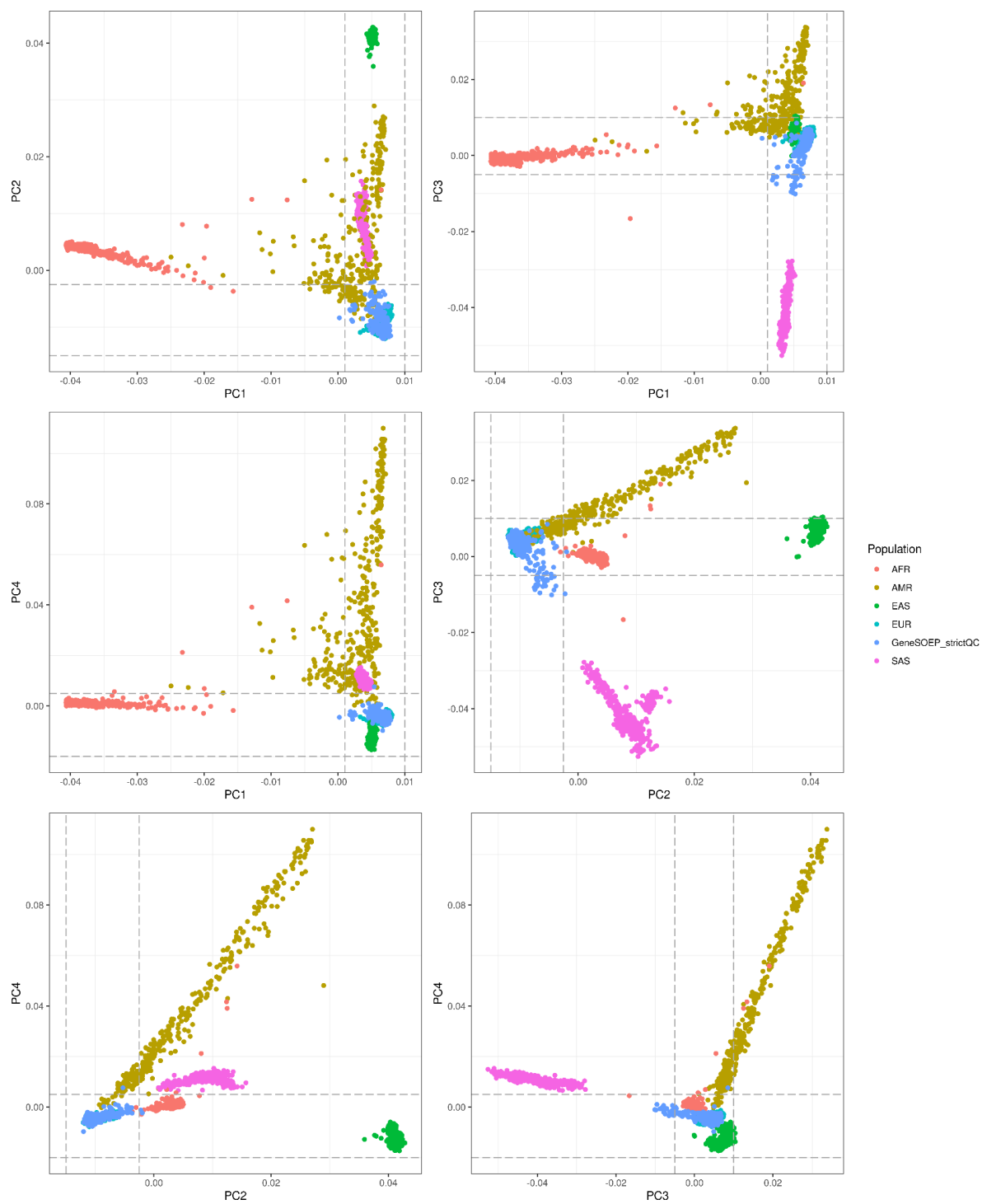

Supplement: S11 Fig — (PDF) [file pone.0294896.s011.pdf]

**S12 Fig.** Flow-chart quality control of genetic data

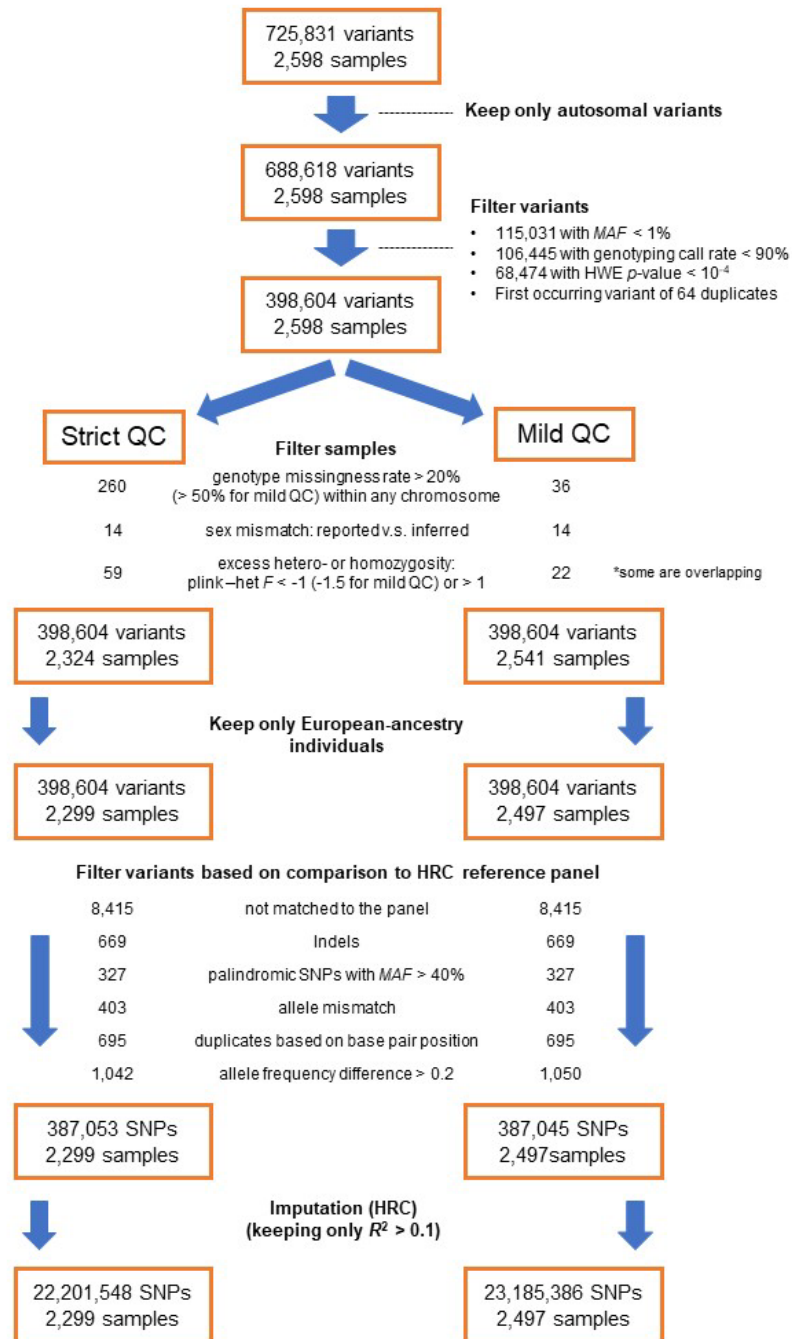

Supplement: S12 Fig — (PDF) [file pone.0294896.s012.pdf]
