# Supplementary material for: Cohort profile: Genetic data in the German Socio-Economic Panel Innovation Sample (SOEP-G)
Source: PLoS One. 2023 Nov 29;18(11):e0294896. doi: 10.1371/journal.pone.0294896 (PMC10686514; doi:10.1371/journal.pone.0294896)
Supplement: S2 Fig — (PDF) [file pone.0294896.s002.pdf]

S2 Fig. Histogram of genotype call rates of SNPs and samples

A. SNP call rates

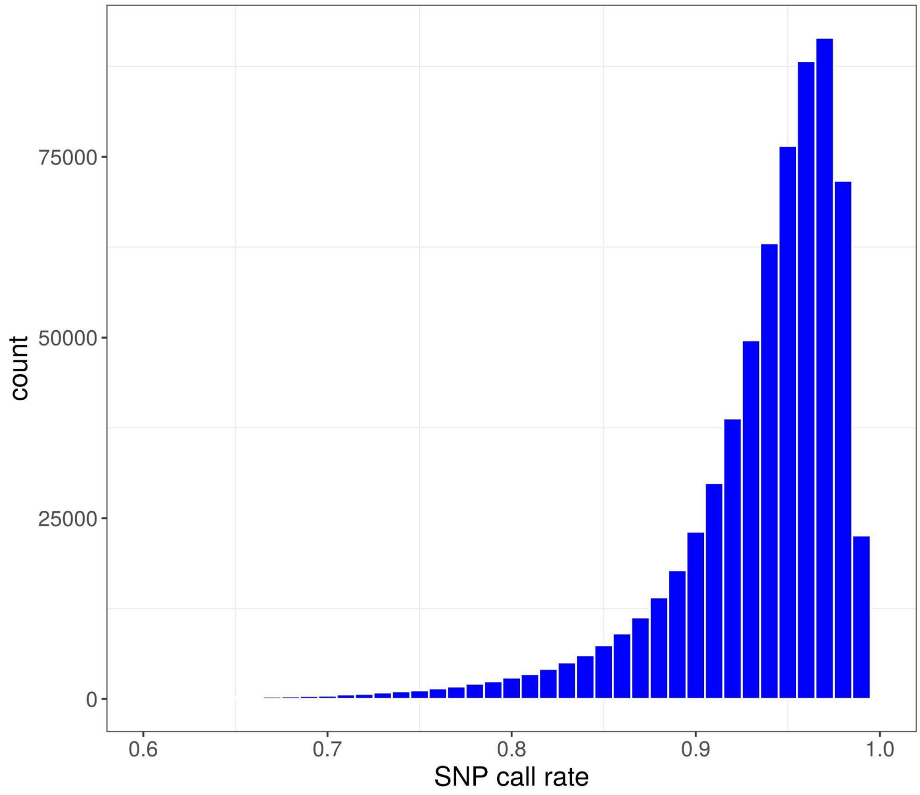

B. Sample call rates

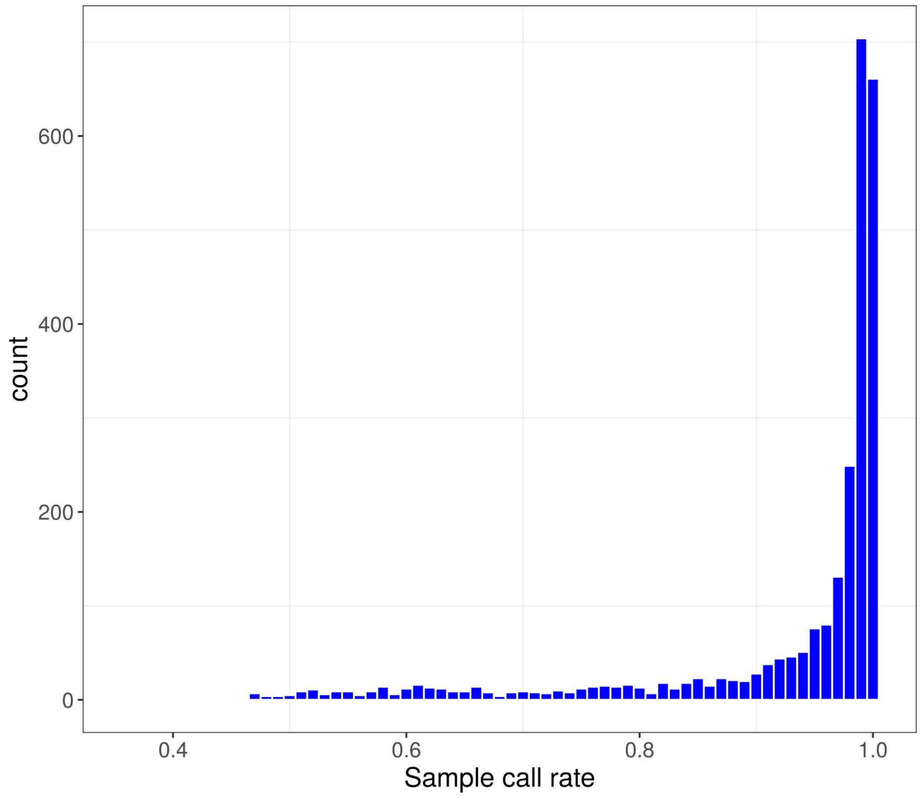

Note: only autosomal biallelic SNPs were used.
