# Supplementary material for: Cohort profile: Genetic data in the German Socio-Economic Panel Innovation Sample (SOEP-G)
Source: PLoS One. 2023 Nov 29;18(11):e0294896. doi: 10.1371/journal.pone.0294896 (PMC10686514; doi:10.1371/journal.pone.0294896)
Supplement: Attachment — Submitted filename: Response letter.docx [file pone.0294896.s021.docx]

**Contents**

1. **Editor/Reviewer comments and response letter for the first round revision**
2. **Editorial requests after the revise-and-resubmission and responses**

**1. Editor/Reviewer comments and response letter for the first round revision**

**Editor:**

Thank you for submitting your manuscript to PLOS ONE. After careful consideration, we feel that it has merit but does not fully meet PLOS ONE’s publication criteria as it currently stands. Therefore, we invite you to submit a revised version of the manuscript that addresses the points raised during the review process.

**Reviewer 1:**

**This paper provides a detailed description of the Gene-SOEP cohort. The text is well written and descriptive. The paper will surely be useful and well appreciated by current and future users of the SOEP resource as well as readers of papers based on that dataset. Below are few comments and questions to the authors that I believe should be addressed.**

We appreciate the positive remarks and suggestions by the reviewer.

**1) The authors claim in the abstract and many times throughout the paper that that the Gene-SOEP is “representative of the entire German population” and list that as one of the major strengths of the sample in their discussion section.**

**It is not clear to me how one could make such a strong claim and I believe, if not clarified further, this claim could proof misleading for future users of the Gene-SOEP, that will most surely cite this paper.**

**The sample is a very small fraction of the German population and the participation in Gene- SOEP was optional. Indeed, as the authors point out on page 9, only 58% of the invited adults consented to participate in the Gene-SOEP. Also, on page 8 the authors note that residents without German citizenship were under-represented in the sample which indicates that the sample is subject to participation bias.**

**As this is a genetic dataset, one would assume that the authors are implying, with their claims of representativeness, that the sample is representative of the German population in terms of allele frequencies. An unusual assumption given the size of the sample, the participation rate and what previous studies have shown about the genetic component of study engagement among participants in genetic studies (for example doi:10.1038/s41467-021-21073-y and doi:10.1093/ije/dyy060).**

**Do the authors believe that the sample allele frequencies in the Gene-SOEP cohort are representative of the population allele frequencies in the German population?**

We tuned down the language about the representative nature of the sample throughout the revised paper. Specifically, we state that *SOEP-G [throughout the manuscript, we have changed the acronym to align it with other SOEP acronyms, e.g. SOEP-IS, the "Innovatios Sample" of SOEP or SOEP-core] is based on a representative sample of households in Germany* and demonstrate similarities and differences between SOEP-G and the much larger SOEP-core sample. We also added a clarification on what we mean by representativeness: *representativeness means that all groups of persons are covered by the survey Unequal participation rates are compensated for in descriptive figures by weighting the cases accordingly; weighting is not necessary for analyses of subgroups with different sampling probabilities and for multivariate analyses. Please see below for our related response.*

**2) The authors note on page 8 that compared with census data, the Gene-SOEP sample is very similar to the German population in terms of age, sex and living region but that residents without German citizenship are under-represented in the Gene-SOEP sample. Is it possible for the authors to explore more phenotypes in this manner?**

We added additional phenotypes (alcohol consumption, smoking, height, and BMI) in Table 1 as well as parental information in Table 2 (age at birth, education, and income). We also added information from the SOEP-core sample. In the revised text, we added: *"However, the comparison to the overall SOEP-core sample shows that the participants in the SOEP-IS sample are overall socioeconomically better-off than those in the core sample and potentially the overall German population*”.

**3) From page 21: “Interestingly, Figure 4 also demonstrates that younger birth cohorts are on average substantially taller than older birth cohorts.” Many studies have explored the height trend of birth cohorts (e.g. see** [**https://doi.org/10.7554/eLife.13410.001**](https://doi.org/10.7554/eLife.13410.001)**) Is this result consistent with what would have been expected from previous studies?**

We added the suggested reference and edited the sentence to: “*Consistent with the previous findings*[*^64^*](https://paperpile.com/c/wP8wuO/v6vv)*, Interestingly, Figure 4 also demonstrates that younger birth cohorts are on average substantially taller than older birth cohorts.*”

**4) On page 3 the authors say: “The heritabilities of behavioral, psychological, and economic phenotypes (e.g. educational attainment, personality, risk attitudes) and health outcomes (e.g. cardiovascular disease, dementia) are typically between 30% and 70%, with an average heritability of 49% across all traits.” Rather strong wording here as the authors are referring to estimates. Further: a) Are these heritability estimates based on twin studies? b) Do they comply with studies that estimate heritability in a different manner?**

We clarified that these estimates are based on twin studies. Twin-based heritability estimates tend to be much larger than heritability estimates based on molecular genetic data. Since it is beyond the scope to discuss this missing heritability concept, we referred to relevant papers for readers who may be interested. We also added the standard error for the average heritability estimate.

**5) It would be useful for the reader if you were to add reference for the following claim on page 15: “The effect sizes of individual single nucleotide polymorphisms (SNPs) on behavioural traits and complex diseases are usually tiny (R2 < 0.05%).”**

We added a reference to Okbay et al. 2016 (10.1038/nature17671).

**6) On page 14 the authors write: “approximately independent SNPs”. What does approximately independent mean here in terms of pairwise LD?**

The details are provided in Section 5 of Supplementary Information. We pruned the remaining SNPs using an cutoff of 0.01 and a 1Mb rolling window incremented in steps of 5 variants in Plink. We added *“(pairwise r^2^<0.01)”* in the sentence to make it more clear and added “*(see Supplementary Information section 5)*”.

**Reviewer 2:**

**This paper describes the collection of genetic data from 2,598 participants in the German Socio-Economic Panel (SOEP) and the construction of 66 polygenic indices (PGIs) that are made available for analyses. It also presents some findings using these data, related to how well the PGIs predict phenotypes in the data, cohort trends in both PGIs and phenotypes, and associations between self-reported health and different PGIs.**

**This is a clear and well-written paper that will be useful to make the research community aware of this novel source of genetic data. As described in the paper, SOEP contains very rich and longitudinal information on respondents and the addition of genetic data is extremely welcomed. One particular strength of the data is the inclusion of 107 full-sibling pairs, 501 parent-offspring pairs and 152 parent offspring trios which allow researchers to exploit family-based research designs and to isolate random variation in PGIs due to Mendelian inheritance. The paper is already in good shape and my main suggestion for improvement is to add some simple results related to the family data.**

We thank the reviewer for the positive remarks and helpful suggestions.

**1) Since using genetic variation from family data is currently in the frontier of research in social- science genetics, it would be useful to present a simple exercise in this paper to exemplify how family data can be used and why that is interesting (i.e. because it allows researchers to exploit random variation in genetics). This would be especially useful for applied researchers who have not used this type of data before. Using the sample for which parents PGI is available (or can be imputed from sibling information) one could show results of a participant's PGI on her outcomes conditional on parents' PGI. It would be interesting to show in this sample that the random component of the PGI(s) is uncorrelated with a range of variables determined before birth (a "balance test" for randomization using characteristics such as parents' education and place of birth) but that it causally affects the participant's adult outcomes such as SES and health. Given the wealth of information in the SOEP I'd imagine there would be no shortage of predetermined and outcome characteristics to explore. Adding this would highlight the strength of analyzing family genetic data and help promote the Gene-SOEP data.**

We agree with the referee that it would be interesting to demonstrate an example of family-based analysis. As suggested, we examined the correlation between offspring PGI for EA and parental phenotypes (age at birth, education, and income) while conditioning on paternal and maternal PGI for EA. We indeed found that the random component of the offspring PGI is uncorrelated with parental phenotypes. In the interest of the overall flow of the manuscript, we reported this analysis in Section 8 of the Supplementary Information.

**2) While tables 1 and 2 are informative it seems like more could be done to document selection into the sample. For example, how do phenotypes for which PGIs are constructed compare between the full SOEP-IS sample and the Gene-SOEP sample (or between those who were invited but ended up not genotyped and those who were genotyped)?**

We added additional phenotypes (alcohol consumption, smoking, height, and BMI) in Table 1 as well as parental information in Table 2 (age at birth, education, and income). We also added information from the SOEP-core sample. In the revised text, we added: *"However, the comparison to the overall SOEP-core sample shows that the participants in the SOEP-IS sample are overall socioeconomically better-off than those in the core sample and potentially the overall German population*”. As far as the IS sample is concerned, we do not observe the difference between those genotyped and those not genotyped. We also added a clarification on what we mean by representativeness: representativeness means that all groups of persons are covered by the survey. Unequal participation rates are compensated for in descriptive figures by weighting the cases accordingly; weighting is not necessary for analyses of subgroups with different sampling probabilities and for multivariate analyses.

**3) The discussion about QC on pages 12 and 13 are quite technical and use a lot of unexplained jargon (example: call rates). There is room for making such discussion clearer.**

Throughout this section, we added explanations to such jargons or replaced them with easier terms (for instance, “*genotype missingness rate was greater than 5%”* instead of *“call rates were smaller than 95%”*).

**4) The result on page 23/figure 5 that the higher BMI for older birth-cohorts cannot be explained by aging contradict the evidence of the sharp rise in obesity during this time period. I am not familiar with the German context, but in the US one would expect BMI to the larger for younger cohorts once age is controlled for.**

Our claim is that aging by itself cannot explain the higher average BMI in the older birth cohorts. We do not claim that aging is not a factor. We clarified this as follows: *“Thus, the higher BMI in the older birth-cohorts is likely to be caused by a combination of environmental effects such as differences in living conditions, socio-economic effects,*[*^68^*](https://paperpile.com/c/wP8wuO/4eO6h) *or nutrition*[*^69^*](https://paperpile.com/c/wP8wuO/LuM71) *in addition to general effects from aging.”*

**2. Editorial requests after the revise-and-resubmission**

**We've checked your submission and before we can proceed, we need you to address the following issues:**

**1) Please provide additional details regarding participant consent. In the ethics statement in the Methods and online submission information, please ensure that you have specified what type you obtained (for instance, written or verbal, and if verbal, how it was documented and witnessed). If your study included minors, state whether you obtained consent from parents or guardians. If the need for consent was waived by the ethics committee, please include this information.**

We added sentences that stated this was a written form.

**2) Please note that funding information should not appear in the Acknowledgments section or other areas of your manuscript. We will only publish funding information present in the Funding Statement section of the online submission form. Please remove any funding-related text from the manuscript and let us know how you would like to update your Funding Statement. Currently, your Funding Statement reads as follows: The data collection was supported through the German Research Foundation (Leibniz Prize to RH) - https://www.dfg.de/en/, a European Research Council Consolidator Grant (647648 EdGe to PK) - https://erc.europa.eu/, the Jacobs Foundation (EMTD, KPH, DWB) - https://jacobsfoundation.org/en/, NIH/NICHD grant R01HD092548 (KPH) - https://www.nih.gov/, the NORFACE DIAL Grant 462-16-100 (PB) - https://www.norface.net/, the University of Basel (RM) - https://www.unibas.ch/en.html, and the Canadian Institute for Advanced Research (DWB) - https://cifar.ca/. The Population Research Center at the University of Texas at Austin (KPH and ETD) is funded by NIH center grant P2CHD04284. The funders had no role in study design, data collection and analysis, decision to publish, or preparation of the manuscript.”**

We removed the funding section. The previous funding statements were included in the online submission form as they are.

**3) We note that Figure 2 in your submission contain [map/satellite] images which may be copyrighted. All PLOS content is published under the Creative Commons Attribution License (CC BY 4.0), which means that the manuscript, images, and Supporting Information files will be freely available online, and any third party is permitted to access, download, copy, distribute, and use these materials in any way, even commercially, with proper attribution. For these reasons, we cannot publish previously copyrighted maps or satellite images created using proprietary data, such as Google software (Google Maps, Street View, and Earth). For more information, see our copyright guidelines:** [**http://journals.plos.org/plosone/s/licenses-and-copyright**](http://journals.plos.org/plosone/s/licenses-and-copyright)**.**

**We require you to either (1) present written permission from the copyright holder to publish these figures specifically under the CC BY 4.0 license, or (2) remove the figures from your submission:**

We added the data source and the license as required by the data provider.

**4) Please include captions for your Supporting Information files at the end of your manuscript, and update any in-text citations to match accordingly. Please see our Supporting Information guidelines for more information:** [**http://journals.plos.org/plosone/s/supporting-information**](http://journals.plos.org/plosone/s/supporting-information)**.**

We added a caption for the supplementary text

**5) Your ethics statement should only appear in the Methods section of your manuscript. If your ethics statement is written in any section besides the Methods, please move it to the Methods section and delete it from any other section. Please ensure that your ethics statement is included in your manuscript, as the ethics statement entered into the online submission form will not be published alongside your manuscript.**

We had ethics statements in the Acknowledgement section. These were moved to the beginning of the Method section.
